# Long-Term Tumor Control After Carbon Ion Radiation Therapy Boost in Locally Advanced Cervical Clear Cell Adenocarcinoma

**DOI:** 10.1016/j.ijpt.2025.101200

**Published:** 2025-08-14

**Authors:** Amelia Barcellini, Alessandro Vai, Eloisa Arbustini, Marco Carnelli, Sara Imparato, Durim Delishaj, Carlo Pietro Soatti, Carmine Tinelli, Elisabetta Vitali, Viviana Vitolo, Ester Orlandi

**Affiliations:** 1 Department of Internal Medicine and Therapeutics, University of Pavia, Pavia, Italy; 2 Radiation Oncology Unit, Clinical Department, CNAO National Center for Oncological Hadrontherapy, Pavia, Italy; 3 Medical Physics Unit, Clinical Department, CNAO National Center for Oncological Hadrontherapy, Pavia, Italy; 4 Centre for Inherited Diseases, Department of Research, Fondazione IRCCS Policlinico San Matteo, Pavia, Italy; 5 Gynecology and Obstetrics Unit, ASST Papa Giovanni XXIII, Bergamo, Italy; 6 Radiology Unit, Clinical Department, CNAO National Center for Oncological Hadrontherapy, Pavia, Italy; 7 Department of Radiation Oncology, Alessandro Manzoni Hospital, Lecco, Italy; 8 CNAO National Center for Oncological Hadrontherapy, Pavia, Italy; 9 Radiation Oncology Unit, ASST Papa Giovanni XXIII, Bergamo, Italy; 10 Department of Clinical, Surgical, Diagnostic, and Pediatric Sciences, University of Pavia, Pavia, Italy

**Keywords:** Carbon ion radiation therapy, Clear cell adenocarcinoma, Cervix, Abscopal effect

## Abstract

Clear cell adenocarcinoma of the uterine cervix is a rare and aggressive subtype of cervical cancer, typically resistant to conventional radiation therapy and lacking dedicated treatment guidelines. We present the case of a young patient with an ataxia telangiectasia mutation and locally advanced disease, who was unfit for brachytherapy following standard chemoradiotherapy and subsequently received a carbon ion radiation therapy boost. This mixed-beam strategy was well tolerated and led to durable local control along with a nodal response, which is suggestive of a possible abscopal effect. These findings underscore the potential of carbon ion radiation therapy in overcoming radioresistance and suggest a contributory role of genetic background in mediating systemic immune effects.

## Introduction

Clear cell adenocarcinoma of the uterine cervix (CCAC) is a rare and highly invasive histological subtype, accounting for approximately 4% to 9% of all cervical adenocarcinomas. It is the second most common Human Papilloma Virus (HPV)-independent endocervical adenocarcinoma and is characterized by a bimodal age distribution, with incidence peaks around 26 years (range 17-37) and 71 years (range 44-88),^1^ especially in the case of diethylstilbestrol exposure.^2^

Due to the rarity of this disease, available data are limited and primarily derived from retrospective studies or case series. As a result, there are no specific guidelines or consensus statements dedicated to this tumor subtype, and its management generally follows protocols established for more common histotypes of cervical cancer. In cases of locally advanced disease, the standard treatment recommendation is radical radiochemotherapy (RT/CT) followed by brachytherapy (BT).^2^, ^3^, ^4^, ^5^, ^6^ However, CCAC exhibits distinct molecular and cellular features contributing to resistance to conventional radiation therapy (RT).^7^, ^8^, ^9^

In recent years, carbon ion radiation therapy (CIRT) has shown clinical efficacy in treating various radioresistant tumors, and emerging literature supports its potential role in managing locally advanced cervical adenocarcinomas. In addition to their well-known radiobiological advantages over photons in treating radioresistant tumors, carbon ions have also demonstrated greater immunogenicity and the ability to induce abscopal effects.^10^

In this report, we present a case of durable local and nodal response in a young ataxia telangiectasia mutated (ATM) patient with clear cell carcinoma of the cervix who underwent a CIRT boost following standard RT/CT.

## Material and method

To describe our clinical experience, we followed the Checklist for Reporting Case Reports.^11^ An independent ethics committee approved the conduct of this analysis (CNAO OSS 68 2024-Particle GYN on August 13, 2024). Written informed consent was obtained from the patient for research purposes.

## Case report

### Oncologic anamnesis, clinical-radiologic findings, and treatments

**Figure 1:**
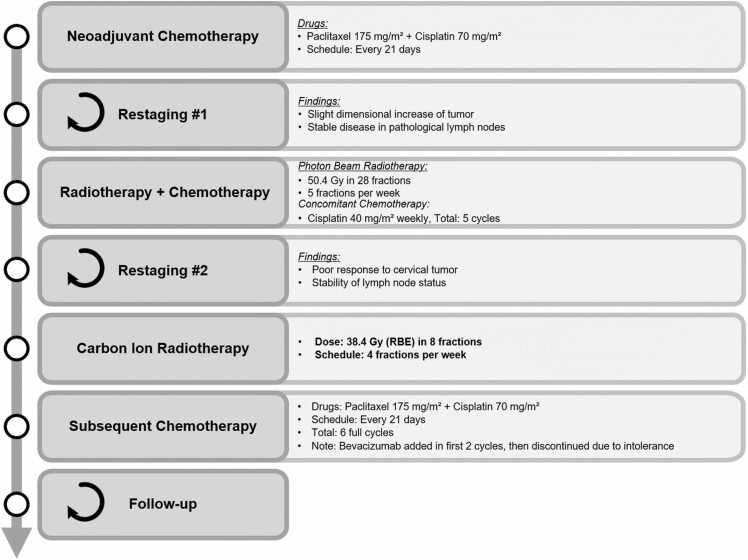
Schematic summary of the patient's therapeutic pathway.

A 33-year-old woman, not exposed to diethylstilbestrol, with a history of endometriosis treated with estrogen-progestin therapy for 18 years, underwent a gynecological evaluation due to persistent episodes of spotting. Clinical assessment revealed a large cervical lesion involving the left parametrium and extending to the pelvic wall, as well as the involvement of the posterior and right parametria. Biopsy results were diagnostic for grade 3 clear cell adenocarcinoma of the cervix, with a Ki67 of 80% to 90%. Subsequent staging confirmed local regional extension (later-lateral dimension of 64 mm, anteroposterior dimension of 42 mm, and cranio-caudal dimension of 52 mm). Moreover, it revealed multiple pathological lymph nodes along both iliac axes and in the interaortocaval region. Figure 1 schematically summarizes the patient's therapeutic pathway.

**Figure 2:**
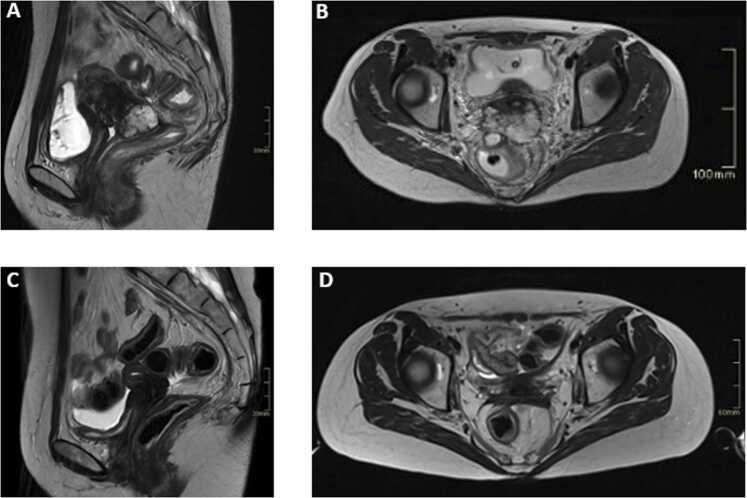
Magnetic resonance imaging (MRI) of the pelvic lesion before and after carbon ion radiation therapy (CIRT) boost. (A) and (B) Pretreatment MRI in the sagittal (A) and axial (B) planes shows a pathological mass centered at the level of the uterine cervix, infiltrating the posterior vaginal fornix and the posterior wall of the upper third of the vagina. The lesion demonstrates multilobulated margins with nodular extensions, including an anterior nodule invading 15 mm into the right parametrium. Posteriorly, the mass infiltrates the anterior perirectal fat tissue with a distance of 2 mm from the rectum. An exception is noted for a pseudo-nodular extension that comes into close contact with the anterior wall of the proximal rectum, with no clearly discernible intervening fat plane. (C) and (D) Post-treatment MRI in the sagittal (C) and axial (D) planes obtained 6 months after the CIRT boost demonstrates a complete response.

After a multidisciplinary case discussion, the patient was considered for neoadjuvant treatment with paclitaxel 175 mg/mq and cisplatin 70 mg/mq for downsizing to perform a subsequent surgery. However, magnetic resonance imaging (MRI) after the second cycle showed a slight dimensional increase in the cervical lesion. The patient then underwent conventional pelvic RT (total dose of 50.4 Gy delivered in 28 fractions, 5 fractions per week), including the interaortocaval region, combined with weekly cisplatin chemotherapy. At the clinical and instrumental reassessment at the end of the RT/CT treatment, necessary to evaluate the technical feasibility of BT boost, the cervical lesion showed a poor response on the cervical tumor (later-lateral dimension of 67 mm, anteroposterior dimension of 34 mm, and cranio-caudal dimension of 51 mm), with a stability of the lymph node status **(**Figure 2A and B).

A radiation oncologist with over 10 years of expertise in BT considered that a BT boost would not provide optimal coverage for the treatment target. Additionally, an expert in stereotactic treatments judged a stereotactic body RT (SBRT) boost as suboptimal, considering the poor response to the prior RT, the close proximity to the rectum and the previous doses.

In light of the patient's young age, the limited response to the initial phase of treatment, and the known radioresistance of the disease, as well as the lack of indication for a BT boost, the case was reviewed in a multidisciplinary setting. Considering the availability of alternative therapeutic options, the multidisciplinary team deemed a carbon ion boost as the most appropriate therapeutic strategy, followed by CT.

### Carbon ion radiation therapy as a boost

**Figure 3:**
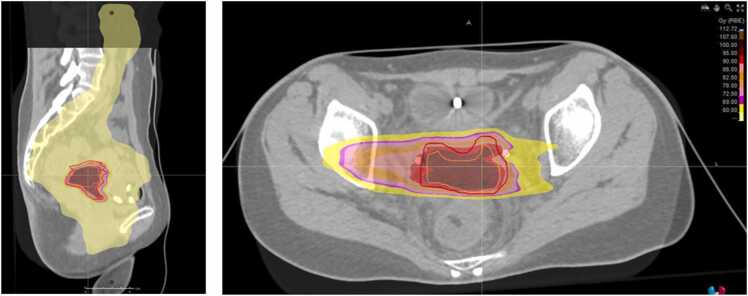
Summed dose distribution of the 2 treatment plans (dose from external beam photon radiation therapy and carbon ion radiation therapy - CIRT), expressed in EQD2. The photon beam plan was prescribed at 95% of the volume, while CIRT was at 50% of the volume. The nominal prescribed median total dose to the clinical target volume (CTV) exceeded 94.8 Gy(RBE). Due to the proximity of critical organs at risk, the dose was reduced in the cranial portion of the CTV. Note that the two planning CT scans were registered using different patient setup conditions.

The patient was then treated with CIRT boost, delivering a total dose of 38.4 GyE in 8 fractions, with 4 fractions per week using the intensity-modulated particle therapy. Given that BT was considered not feasible, CIRT was employed as a boost specifically targeting the cervical tumor. Consequently, no boost was administered to the involved lymph node sites, which were outside the high-dose volume. The treatment plan included 2 fixed horizontal beams, robustly optimized for a set of uncertainty scenarios (isocenter shift: ±3-5 mm, range uncertainties: ±3.5%) to ensure target coverage and minimize dose to relevant organs at risk near it. The biologically effective dose was calculated using the local effect model I. In preparation for the CIRT treatment, and considering the local extent of the disease, 2 ureteral stents were placed, and the patient was informed about the increased risk of ureteral toxicity. After the first week of treatment, the therapy was temporarily suspended due to grade (G) 3 anemia, likely related to prior RT/CT. Figure 3 shows the summed dose of the 2 treatment plans.

Following the completion of the CIRT treatment, the patient continued with chemotherapy with Paclitaxel 175 mg/mq and Cisplatin 70 mg/mq, of which 6 full cycles were administered; in the first 2, Bevacizumab was also given but was discontinued due to intolerance.

### Genetic assessment

**Figure 4:**
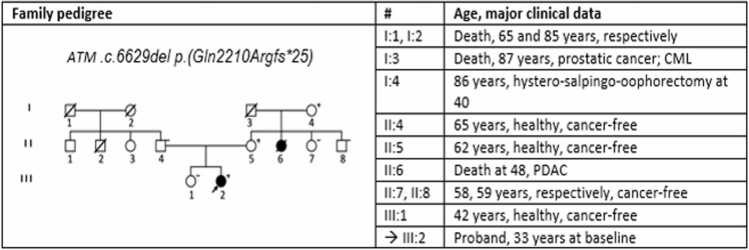
Pedigree of the family. The arrow points to the proband. **+** = carrier of the ATM gene variant; **- =** non-carrier of the ATM gene variant**; CML** = Chronic myeloid leukemia; **PDAC**= pancreatic ductal adenocarcinoma

In light of the patient's clinical history and intending to optimize the following systemic treatment, the patient underwent constitutional genetic testing through an NGS multigene panel (Illumina TruSight Cancer) comprising 94 genes. This investigation revealed the presence of a likely pathogenic heterozygous frameshift variant in the ATM gene, exon 46 [cytoband: 11q22.3c.6629del p.(Gln2210Argfs*25)]. The variant predicts early protein synthesis disruption. Family screening showed that this variant was inherited from the mother and maternal grandmother. A maternal aunt had died at age 48 from pancreatic ductal adenocarcinoma (PDAC) **(**Figure 4).

### Oncological follow-up

The first RT follow-up was conducted at the particle beam center approximately 6 weeks after the completion of the CIRT boost. Subsequently, the patient was monitored every 3 to 4 months for the first 2 years, and then every 6 months with joint gynecological and hadrontherapy follow-up.

**Figure 5:**
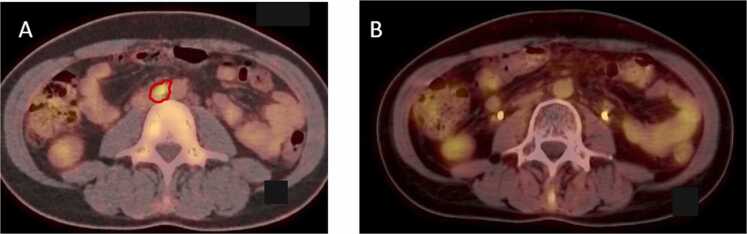
18F-FDG PET/CT images showing metabolic response of para-aortic lymphadenopathy. (A) Pathological hypermetabolic uptake (outlined in red) involving the para-aortic lymphadenopathy, which was progressing after conventional chemoradiotherapy and was not included in the carbon ion boost treatment plan. (B) A complete metabolic response at this site on the first PET scan performed 6 months after the boost.

At the first follow-up, the cervical lesion showed a size reduction (later-lateral dimension of 40 mm, anteroposterior of 10 mm, and cranio-caudal of 19 mm), along with a decrease in the adenopathic conglomerates not irradiated with CIRT, particularly in the interaortocaval lymph node (Figure 5). Six months after CIRT, Fluorodeoxyglucose Positron Emission Tomography (FDG-PET) imaging documented a complete metabolic response in both the cervical lesion and the lymph node metastases. A complete radiological response on MRI was observed at 8 months following CIRT (Figure 2C and D). The patient remained free of local and systemic disease at the time of manuscript preparation (5 years and 6 months post-RT).

Concerning post-treatment toxicities associated with mixed-beam RT, the patient developed G3 ureteral stenosis, as defined by CTCAE v5.0.^12^ Due to recurrent G ≥3 urinary tract infections, the stents were subsequently replaced with bilateral nephrostomies. Additional late toxicities included G1 sacral insufficiency fracture, G1 vaginal atrophy, and G1 vaginal stenosis. Following CIRT, the patient was referred for pelvic floor rehabilitation to mitigate the risk of sexual dysfunction, which was not reported during subsequent follow-up.

## Discussion

This report presents a case of long-term response in a CCAC managed with a multimodal therapeutic strategy comprising mixed-beam RT (photon-based RT combined with a CIRT boost) in conjunction with concurrent and sandwich chemotherapy. We acknowledge the inherent limitations of single-case reports, particularly in terms of the generalizability of findings; however, the novel contributions of this case to the current scientific literature can be outlined as follows:

i) demonstration of the feasibility and potential efficacy of a CIRT boost in radioresistant cervical cancer cases not eligible for BT;

ii) to the best of our knowledge, it may represent one of the first potential abscopal response^13^ following CIRT in cervical cancer;

iii) a hypothesized association between an underlying ATM gene mutation and the response.

A recent analysis of data from the Surveillance, Epidemiology, and End Results database reported significantly improved overall survival with surgical management compared to definitive RT/CT in patients with CCAC known for its intrinsic radioresistance (73.4% vs 35.6%, *P* = .000), even in locally advanced stages (62.5% vs 37.4%, *P* = .000).^5^ However, in this case, given the patient's inoperability, a combined RT/CT regimen was selected as the treatment strategy.

BT is considered a standard component in the definitive RT for locally advanced cervical cancer^14^; however, it is noteworthy that 15% to 20% of patients may be deemed ineligible for this modality due to anatomical issues, technical limitations, comorbidities, or refusal of the procedure.^15^, ^16^, ^17^, ^18^, ^19^ Additionally, limited access to BT in resource-constrained settings remains a major concern.^20^ When BT is not feasible, SBRT has been explored as an alternative boost modality. However, the total deliverable dose is often constrained by prior exposure to adjacent organs at risk.^17^, ^19^ The advantageous ballistic properties of particle therapy, particularly carbon ions, allow for maximal dose delivery to the target while minimizing exposure to surrounding healthy tissues, thereby overcoming the limitations of SBRT—an approach that proved successful in our experience.

Tumor volume does not appear to be significantly associated with RT response for locally advanced adenocarcinoma; indeed, for equivalent volumes, adenocarcinomas exhibit a lower probability of local control compared to squamous cell carcinomas when treated with definitive RT.^21^, ^22^ These findings suggest that factors beyond radiation dose are critical for improving LC in this setting. In this context, CIRT, compared to hypofractionated photon-based treatments, demonstrates enhanced effectiveness in overcoming radioresistance, making it an optimal tool in challenging cases such as this one, where achieving durable disease control is critical.

A recent Japanese multicenter prospective registry study on chemo-CIRT for locally advanced cervical adenocarcinoma reported a 2-year LC rate of approximately 81%,^23^ notably higher than the 70% to 74% reported for conventional RT/CT.^24^, ^25^, ^26^, ^27^, ^28^ Furthermore, 2-year overall survival rates with chemo-CIRT ranged from 88% to 98%,^23^, ^29^, ^30^ substantially exceeding those of historical standard photon beam RT cohorts.

In addition to physical dose advantages, densely ionizing RT, such as carbon ions, may confer biological benefits by triggering distinct cell death pathways and promoting the release of inflammatory cytokines.^31^ These factors can potentially stimulate a systemic immune response, leading to the elimination of distant, nonirradiated metastatic lesions—a phenomenon known as the abscopal effect.^32^, ^33^ Although rare, the abscopal effect has been previously documented in both preclinical and clinical studies involving CIRT.^34^, ^35^, ^36^ In our case, the para-aortic pathological lymph nodes, which had been unresponsive to prior RT/CT and did not receive either a photon boost or direct CIRT irradiation, began to regress significantly after CIRT and ultimately achieved a durable complete response. While this observation may suggest a potential abscopal effect, it is also plausible that the immunogenic properties of CIRT contributed to enhancing tumor sensitivity to the subsequent systemic therapy, which had previously been ineffective.^13^

It is plausible that the patient's ATM mutation contributed to this phenomenon. Mutations in the ATM gene are associated with marked radiosensitivity due to impaired chromosome rejoining—5 to 6 times less efficient than in normal cells, and reduced efficiency in repairing DNA double-strand breaks.^37^ Their inability to arrest cell cycle progression leads to the characteristic Radiation Resistant Synthesis (RDS) phenotype.^38^, ^39^ Consequently, ATM-deficient tumors might be more susceptible to RT. Moreover, ATM inhibition in normal cells has been associated with severe adverse effects, as well as homozygous individuals exhibiting a predisposition to early-onset cancers and severe adverse reactions to conventional RT doses. Heterozygotes, while less severely affected, have an increased risk of malignancy and display some degree of hypersensitivity to ionizing radiation in in vitro studies.^37^, ^40^, ^41^, ^42^ Although the patient carried a genetic predisposition, no notable response to photon-based RT was observed. It is possible that the increased sensitivity associated with the ATM variant may have been enhanced by the higher biological effectiveness of CIRT, potentially contributing to the observed abscopal response. However, further mechanistic studies are needed to substantiate this potential association.

In addition a recent Dutch nationwide study investigated the prevalence of germline pathogenic variants in 25 cancer susceptibility genes among 473 patients with PDAC and extra-pancreatic malignancies. Pathogenic variants were identified in 75 (16%) patients, with ATM gene variants being the most common (22/75, 29%).^43^ Although the 62-year-old mother of our proband (Figure X, II:5) has remained cancer-free, her sister (untested, Figure X, II:6) died at the age of 48 from PDAC. Consequently, we wonder whether our patient, as well as her mother and grandmother (now 86 years old), merits enhanced PDAC surveillance.

## Conclusion

A mixed-beam RT approach including a CIRT boost in CCAC has proven to be feasible and safe in our experience, achieving long-term local control in a cancer histotype known for its radioresistance. The presence of an ATM mutation may account for the observed nodal abscopal response. Given the impracticality of conducting clinical trials in patients with such rare subtypes, the establishment of a dedicated registry is warranted, with the prospective collection of clinical management data and patient outcomes.

## Funding

Research on oncogenetics is supported by IRCCS Fondazione Policlinico San Matteo, grant 741-rcr2013-71.

## Declaration of Conflicts of Interest

Prof. Ester Orlandi, MD, is an Associate Editor of the *International Journal of Particle Therapy*. The authors declare that they have no known competing financial interests or personal relationships that could have appeared to influence the work reported in this paper.
